# Bio-Inspired Neural Network Dynamics-Aware Reinforcement Learning for Spiking Neural Network

**DOI:** 10.3390/biomimetics11010047

**Published:** 2026-01-07

**Authors:** Yu Zheng, Jingfeng Xue, Junhan Yang, Yanjun Zhang

**Affiliations:** School of Computer Science, Beijing Institute of Technology, Beijing 100081, China

**Keywords:** bio-inspired, Spiking Neural Network, reinforcement learning, neural network dynamics

## Abstract

Artificial Intelligence (AI) has seen rapid advancements in recent times, finding applications across various sectors and achieving notable successes. However, current AI models based on Deep Convolutional Neural Networks (DNNs) face numerous challenges, particularly a lack of interpretability, which severely restricts their future potential. Spiking Neural Networks (SNNs), considered the third generation of Artificial Neural Networks (ANNs), are at the forefront of brain-inspired AI research. The resemblance between SNNs and biological neural networks offers the potential to create more human-like AI systems with enhanced interpretability, paving the way for more trustworthy AI implementations. Despite this promise, the absence of efficient training methods for large-scale and complex SNNs hampers their broader application. This paper investigates bio-inspired reinforcement learning strategies by examining neural network dynamics during SNN training. The aim is to improve learning efficiency and effectiveness for extensive and intricate SNNs. Our findings suggest that using reinforcement learning to focus on neural network dynamics may be a promising approach for developing learning algorithms for future large-scale SNNs.

## 1. Introduction

In recent years, the field of Artificial Intelligence (AI), particularly with Artificial Neural Networks (ANNs), has seen rapid advancements and has been implemented across a range of domains. The predominant form of ANN today is the Deep Convolutional Neural Networks (DNNs). While there have been significant achievements, numerous challenges have arisen as well. Developers are frequently faced with several bottleneck issues [1,2]. For instance, as the depth of DNNs increases, the complexity of their structure grows exponentially, and their interpretability diminishes [3,4]. Consequently, DNN-based AI systems often become enigmatic “black boxes,” posing risks such as unreliable decisions, biases, and privacy concerns.

To mitigate the possible negative impacts of AI and to foster the progress of AI development and its applications, it’s essential to boost public confidence in AI methodologies. To create AI that can be trusted, we must comprehend how AI arrives at its decisions or, at the very least, be able to interpret the outcomes or judgments provided by AI. Nonetheless, given that the learning process of contemporary mainstream deep neural networks is often a “black box,” meeting this requirement is particularly challenging.

We believe that to create AI that is trustworthy, artificial neural networks (ANNs) should operate in a manner more akin to biological neural networks. From a neuroscience standpoint, the capability of the human brain to manage a wide array of tasks can be linked to three main elements: (1) extensive connectivity; (2) a functional hierarchy; and (3) interneuronal signaling driven by membrane potentials and neurotransmitters, which can be summarized as neural network dynamics [5].

DNNs, or Deep Neural Networks, are designed with inspiration from the hierarchical organization of the biological brain. Despite this inspiration, the fundamental architecture of DNNs differs significantly from biological neuronal networks in terms of neuronal characteristics, which makes it challenging for DNNs to replicate many of the unique mechanisms found in the biological brain.

Research in biology suggests that the human brain might employ learning strategies that differ significantly from traditional artificial neural networks (ANNs) in adjusting the connections among hundreds of millions of neurons. These strategies could enhance performance and efficiency, enabling the human brain to make superior decisions across a broad range of issues in complex and dynamic real-world settings in polynomial time.

Spiking Neural Networks (SNNs) are often considered the third generation of Artificial Neural Networks (ANNs) and currently represent a key area of development in brain-inspired artificial intelligence. In contrast to Convolutional Neural Networks (CNNs) or Deep Neural Networks (DNNs), SNNs bear a closer resemblance to biological neural networks. They use spiking signals to encode, transmit, and process information, with the intensity, timing, and location of these spikes acting as information carriers. This unique feature allows SNNs to handle spatiotemporal data, potentially enhancing the accuracy and efficiency of neural network problem-solving.

In an SNN, the neuron model is a spiking neuron, which deviates from a linear input-output framework. Mimicking the way a biological brain neuron transmits information, the spiking neuron features a membrane voltage and a discharge threshold. It gathers input until the membrane voltage hits this threshold, at which point the neuron emits a spiking signal and resets its membrane voltage to its initial level.

While Spiking Neural Networks (SNN) offer numerous benefits over Deep Neural Networks (DNN), a significant challenge persists in effectively training SNNs. Since computers first emerged in the previous century, researchers have been striving to emulate human cognitive capabilities on these machines. The introduction of the perceptron, multilayer perceptron, and the error Back Propagation (BP) algorithm has significantly contributed to the development of modern Artificial Neural Networks (ANNs) [6]. The BP algorithm works by propagating errors backward from the neural network’s output layer through each preceding layer, enabling neurons at every level to fully leverage the error information. When paired with the gradient descent algorithm to adjust the weights of ANNs, the BP algorithm facilitates the accurate fitting to objective functions [7], forming the backbone of most current ANNs and demonstrating excellent training performances in real-world task validations.

The direct use of combined BP and gradient descent algorithms in SNN is limited due to the difficulty in calculating gradients for SNN. Various algorithms have been proposed to address this issue. Some approaches attempt to employ gradient-like methods, such as SpikeProp [6], though the performance of the SpikeProp algorithm is not always ideal. Other methods are based on biological insights, such as Hebb’s rule [8] or STDP (Spike-Timing Dependent Plasticity) [6,9,10]. These methods offer ways to update SNN synaptic weights without relying on BP and gradient descent algorithms. However, the training efficiency of these biologically-inspired methods is suboptimal for large-scale and complex SNNs.

Reinforcement learning [11] is a type of unsupervised approach where target agents are trained by interacting with the environment and receiving rewards or punishments. This process involves learning an optimal action strategy primarily through searching and estimating during “trial and error.” A problem can be described through the interaction of a series of four-term tuples (State, Action, Reward, Policy) within the Environment.

One advantage of reinforcement learning is its interpretability. Some researchers have investigated the integration of the reinforcement learning mechanism into the training process of SNN [12]. This approach offers a fascinating method to emulate how the human brain learns causal rules from the environment and seeks to develop a strategy for updating synaptic weights in SNNs through the interaction of environment, action, and reward.

This work, drawing inspiration from biological neural networks, seeks to explore the integration of reinforcement learning within the training of spiking neural networks (SNN) by exploring their dynamics. The aim is to improve the learning efficiency and effectiveness for large-scale, complex SNNs, while also enhancing the interpretability of the SNN training process. Our contributions include: (1) Introducing two distinct mechanisms to investigate how reinforcement learning fits into the learning process of SNNs, with a focus on neural network dynamics; (2) Assessing how the design of the state space impacts the reinforcement learning effects in SNNs; (3) Conducting a detailed analysis of the experimental results and providing specific directions for future research on the application of reinforcement learning in SNN training.

## 2. Related Works

Research in brain-inspired AI primarily focuses on spiking neural networks (SNNs). Compared to traditional artificial neural networks (ANNs) like convolutional neural networks (CNNs) or deep neural networks (DNNs), SNNs utilize spiking neurons, making them more akin to biological neural systems. This similarity allows SNNs to more effectively implement certain pre-established rules from biological research, such as Hebbian learning rules and spike-timing-dependent plasticity (STDP) rules.

The spike sequences produced by neurons in SNNs carry both spatial information, indicated by the neuron’s location, and temporal information, shown by the timing of spikes. This combination is not available to traditional ANNs, providing SNNs with the potential to manage tasks with complex spatial-temporal characteristics.

Spiking Neural Networks (SNNs) utilize spiking signals for data transmission, functioning as an event-driven, asynchronous system. The combination of sparse spiking signal emission and a sparse network structure allows SNNs to be significantly more energy-efficient than conventional Artificial Neural Networks (ANNs) when deployed on chips. For instance, the TrueNorth chip, which uses a spiking neuron architecture, demonstrates enhanced performance and substantially lower power consumption—by several orders of magnitude—compared to running the same algorithm on a von Neumann architecture [13].

Nevertheless, these unique characteristics pose challenges for the effective training of SNNs, particularly when dealing with large-scale and intricate networks. A variety of approaches have been proposed to address this issue.

A number of researchers have investigated methods for converting ANNs to SNNs by replacing a pre-trained ANN model with SNN neurons [14,15,16,17,18,19]. In 2021, a method known as tdBN was introduced by Zheng et al., which enables directly-trained SNNs to expand from fewer than 10 layers to 50 layers. This method effectively addresses issues like gradient vanishing or explosion and balances neuron thresholds and inputs to achieve suitable firing rates, utilizing an adapted batch normalization technique [20]. During the same year, Fang et al. examined the gradient and identity map challenges in SNNs, modified the residual block connections, and introduced SEW ResNet, allowing SNN training to extend to 152 layers [21]. Some ANN-to-SNN methods based on transformers have also been proposed. However, these ANN-to-SNN techniques often fall short of fully leveraging the biological similarity attributes of SNNs, which restricts their optimization potential.

Several researchers have attempted to apply methods inspired by biological principles [22,23,24,25,26,27,28,29,30]. However, the localized optimization problem inherent in the STDP rule restricts the scale of trainable SNNs. There is already some initial research exploring the potential of integrating reinforcement learning with SNN training [31,32,33,34].

## 3. Method

### 3.1. Overview

**Figure 1:**
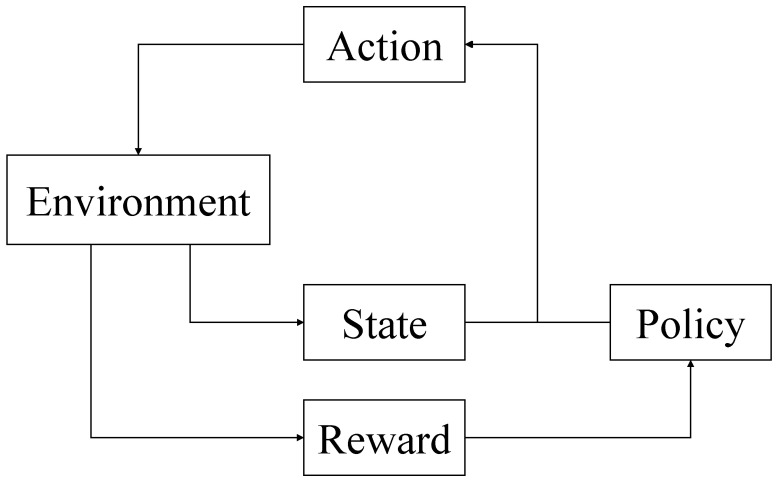
Illustration of reinforcement learning process.

Figure 1 illustrates the process of a typical reinforcement learning method. In reinforcement learning, agents are empowered to discover optimal strategies by interacting with the Environment. The central element in reinforcement learning is the Agent, whose aim is to learn a Policy or a set of Policies that optimize the cumulative rewards obtained from the Environment. As the Environment reacts to the agent’s Actions, it provides feedback, usually through State changes or Rewards. The agent develops the optimal Policy by maximizing these cumulative Rewards. Actions are chosen by the agent based on the Policy concerning the current State [11]. Unlike supervised learning, reinforcement learning’s training process does not need labeled data. During the exploration and assessment of the environment, the agent autonomously tracks the reward level associated with specific actions in particular states, and strives to select actions with higher rewards in future decisions to achieve the ultimate goal of maximizing rewards.

Reinforcement learning can be broadly classified into two main categories: probability-based and value-based [35]. In probability-based reinforcement learning, actions are chosen randomly based on a probability distribution. Throughout the training, the probability of selecting actions that yield higher rewards in a given state is gradually increased, although the action with the highest reward is not always chosen. Conversely, value-based reinforcement learning consistently opts for the action that offers the highest reward. Numerous reinforcement learning techniques have been developed, including Q-Learning [36], Deep Q-Network (DQN) [37], and Policy Gradient [38].

As mentioned earlier, spiking neural networks (SNN) are more akin to biological neural networks. Research in biology indicates that the learning processes within the human brain align remarkably well with reinforcement learning. Furthermore, multiple studies propose the potential existence of reinforcement learning mechanisms in the human brain.

The use of SNN is significantly limited due to the lack of an effective method for training large-scale and complex SNN. Therefore, this paper aims to investigate how reinforcement learning techniques and concepts can be used to efficiently train large-scale and complex SNN.

With this motivation, we proposed two strategies:

- Consider a SNN as a collection of neurons. Therefore, the depiction of a SNN is the result of the collective behavior of numerous agents. As the weight of each synapse is the focal point of training, each synapse is regarded as an agent.
- Regard the SNN as an agent. Implement reinforcement learning techniques on an SNN that has been initially trained using methods similar to backpropagation.

### 3.2. Strategy A: Regard Each Synapse as an Agent

In strategy A, each synapse is regarded as an agent. We employ a reinforcement learning approach to investigate the mechanism for updating synapse weights. We have proposed various state space designs and assessed their impact on the effectiveness of reinforcement learning.

To decrease computational complexity while preserving biological similarity, we chose the LIF (Leaky Integrate-and-Fire) model [39] as the neuron model for our SNN.

As previously mentioned, the reinforcement learning framework consists of four primary components: Action, State, Reward, and Policy. If we consider the synaptic weight update policy among the neurons in the SNN as a policy π to be learned, we can then develop a reinforcement learning model as follows:

#### 3.2.1. Action

The set of actions can involve increasing, decreasing, or maintaining the weight ω of a particular synapse. Given that the synapse’s learning rate is α and the action at time *t* is $a_{t}$, it can then be expressed as:

(1) $$a_{t}\in A=\left\{-\alpha ,0,+\alpha \right\}.$$

The synaptic weight update process could be described as:

(2) $$\omega_{ij}^{k}\leftarrow \omega_{ij}^{k}+\omega_{ij}^{k}*a_{t}$$

#### 3.2.2. State

When designing a strategy for updating synaptic weights, it’s essential to properly design the state space. This involves taking into account the correlation between state *S* and the synapses, which primarily includes the following factors:

- Historical actions and the corresponding rewards: Considering the historical actions along with their corresponding rewards implies that synapses will choose future actions based on the past actions’ effectiveness, potentially introducing past errors into future decision-making. Aman Bhargava’s research incorporates two prior actions and their associated rewards to formulate a learning method, resulting in a learning strategy [12] that is akin to the gradient descent approach. Consequently, our work also factors historical actions and rewards into the state space design process.
- Current synaptic weight: The present synaptic weights hold crucial information to determine the next action in updating synaptic weights. From both computational science and biological perspectives, any learning strategy that neglects the current synaptic weights is very incomplete.
- The topology information of the synapse in the SNN: This is the innovation of our work. To illustrate the logic behind this aspect, let us examine the process of cellular development in biology. Consider, for instance, human embryonic cells. At the outset, the majority of these cells were entirely uniform, yet varying stimuli in different areas led to cellular differentiation. Research in biology suggests that distinct regions of the human brain serve different functions. Excluding location factors fails to account for why brain neurons with identical structures develop varied functions and structures depending on their location.

According to those considerations, we have developed several state space design strategies:

- Model 1: Record two previous actions *a* and corresponding rewards *r*, to construct the state space *S* as:
  (3) $$S=\{a_{t-1},a_{t-2},r_{t-1},r_{t-2}\}$$
- Model 2: Record two previous actions *a* and corresponding rewards *r*, current synaptic weight ω to construct the state space *S* as:
  (4) $$S=\{a_{t-1},a_{t-2},r_{t-1},r_{t-2},\omega \}$$
- Model 3: Record two previous actions *a* and corresponding rewards *r*, current synapse topology location (i,j) to construct the state space *S* as:
  (5) $$S=\{a_{t-1},a_{t-2},r_{t-1},r_{t-2},i,j\}$$
- Model 4: Record two previous actions *a* and corresponding rewards *r*, current synaptic weight ω, current synapse topology location (i,j) to construct the state space *S* as:
  (6) $$S=\{a_{t-1},a_{t-2},r_{t-1},r_{t-2},\omega ,i,j\}$$
- Model 5: Record current synaptic weight ω, current synapse topology location (i,j) to construct the state space *S* as:
  (7) S={ω,i,j}

We will evaluate those state space designs through experiments.

#### 3.2.3. Reward

The reward *r* is designed to be inversely proportional to the loss function of the SNN for the given task. This setup ensures that the reinforcement learning strategy achieves the maximum reward when the loss function of the SNN’s output is at its lowest.

#### 3.2.4. Policy

We will implement the ϵ−greedy policy, in which the likelihood of selecting an action can be expressed as:

(8) $$\pi \left(s_{t}\right)=\left\{\begin{array}{c} argmax_{a\in A}Q\left(s_{t},a\right),Pr=1-\epsilon \\ random-uniform(a\in A),Pr=\epsilon \end{array}\right.$$

Whenever there’s a need to pick an action, the policy strategy either randomly selects an action with a probability of ϵ or chooses the action with the highest *Q* value with a probability of 1−ϵ. In this work, we implement a single-step update strategy. Each time an action is selected or the state undergoes a change, the parameters of the SNN are updated. This process is enhanced by the Experience Replay mechanism, to improve the use efficiency of the training data.

### 3.3. Strategy B: Regard SNN as an Agent

In strategy B, the entire SNN is considered as an agent. This approach is termed DQSN (Deep Q-Spiking neural Network), which uses alternative gradients for backpropagation (BP) in conjunction with reinforcement learning to train the SNN. DQSN mirrors the architecture of DQN, but incorporates spiking neurons and executes alternative gradients for BP. It also employs the ϵ−greedy strategy, a single-step update strategy, an experience replay mechanism, and other similar mechanisms.

The DQSN architecture is composed of three layers: (1) the Input layer, (2) the Hidden layer, and (3) the Output layer. The connections between the input layer and the hidden layer, as well as between the hidden layer and the output layer, are fully interconnected. The complexity of the neural network is adjustable by varying the number of neurons in the hidden layer of the DQSN.

## 4. Experiment Results and Discussion

### 4.1. Experimental Framework

The simulation is executed on Spikingjelly. Table 1 presents the fundamental parameters of the LIF model applied in this work. The gym’s inverted pendulum cartpole-v0 was chosen as the target dataset for assessing the reinforcement learning algorithm. This dataset simulates a reinforcement learning environment that requires keeping a vertical inverted pendulum balanced on a moving cart. The agent must decide between two actions (left or right) based on the four-part state (vehicle position, vehicle speed, swing angle, swing speed) provided by the environment, aiming to keep the pendulum upright for as long as possible.

The SNN we develop consists of three layers: (1) Input layer, (2) Hidden layer, and (3) Output layer. Both the input and output layers are specifically designed for this task. The number of neurons in the hidden layer is determined by the experimental setup. In this work, we utilize the direct coding method.

### 4.2. Decide Learning Rate α and Simulation Time T

To determine the values for the learning rate α and the simulation time *T*, we begin by performing an initial experiment using the model outlined in Equation (3).

We evaluate the learning rates across various orders of magnitude, using test data that includes: 0.1, 0.01, 0.001, and 0.0001. To compare these four groups of data, we set the spiking simulation time to T=16 and configure the hidden layer of the SNN with 16 neurons.

**Figure 2:**
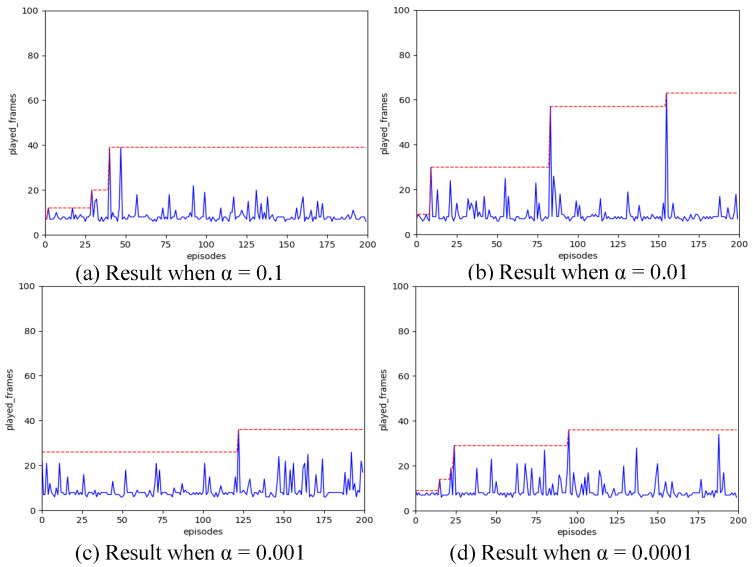
Comparison result of learning rate.

The experimental results depicted in Figure 2 reveal that setting the learning rate α at 0.01 leads to a better evaluation outcome for the SNN network. Decreasing the learning rate further results in changes that are too minimal, hindering effective training. Conversely, increasing the learning rate excessively causes the network parameters to vary too unpredictably, which can easily lead to falling into a local optimum.

To determine the appropriate spiking simulation time *T*, we set the learning rate to 0.01 and configured the hidden layer of the SNN to contain 16 neurons. We then evaluated the performance for *T* values of 4, 8, 16, and 32. The choice of spiking simulation time *T* affects both the performance and the training outcomes of the SNN. If *T* is set too high, it can decrease the operational efficiency of the network. Thus, *T* should be kept as short as possible without compromising the SNN’s spiking distribution effect.

**Figure 3:**
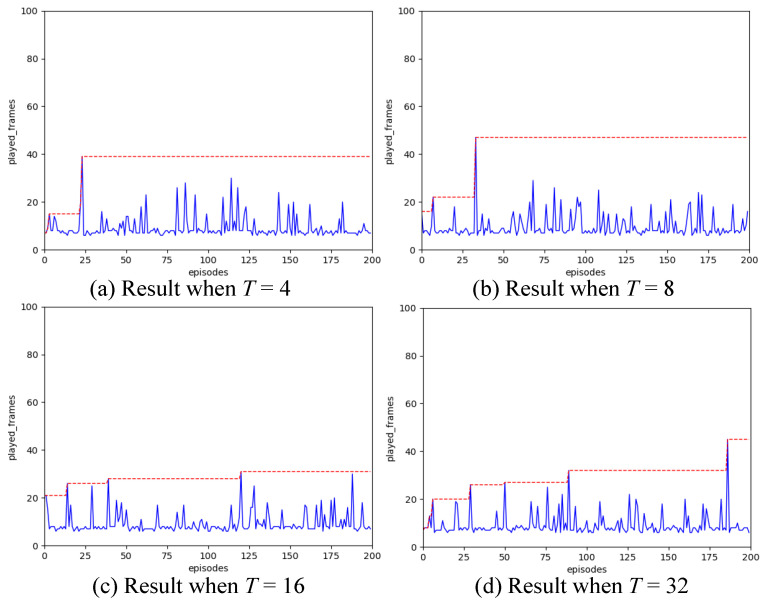
Comparison result of simulation time.

Referring to the experimental outcomes (Figure 3), we can infer that when the duration *T* is too brief, the impact of the SNN network is notably compromised. Conversely, if the duration is excessively long (*T* ≥ 16), the operational efficiency of the SNN diminishes without a significant enhancement in the network effect. Therefore, for the subsequent experiments, the spiking simulation time *T* is established at 16.

### 4.3. Result for Strategy A

**Figure 4:**
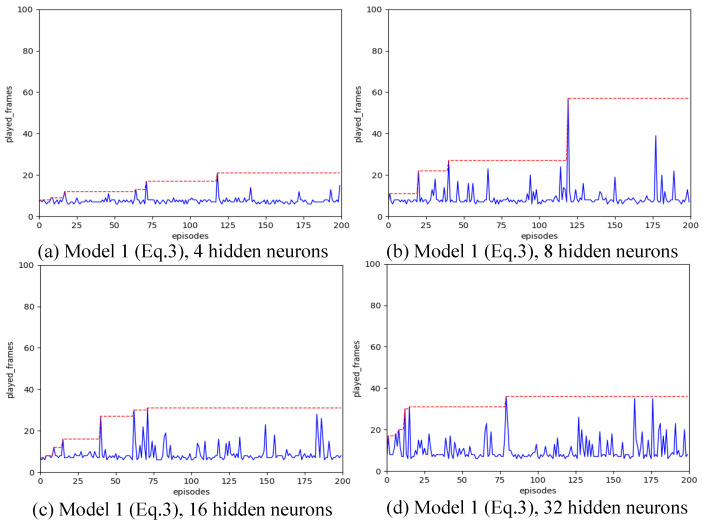
Result for Model 1 with different number of hidden neurons, after increasing the number of hidden layer neurons from 8 to 32, the search power of reinforcement learning algorithms has decreased.

**Figure 5:**
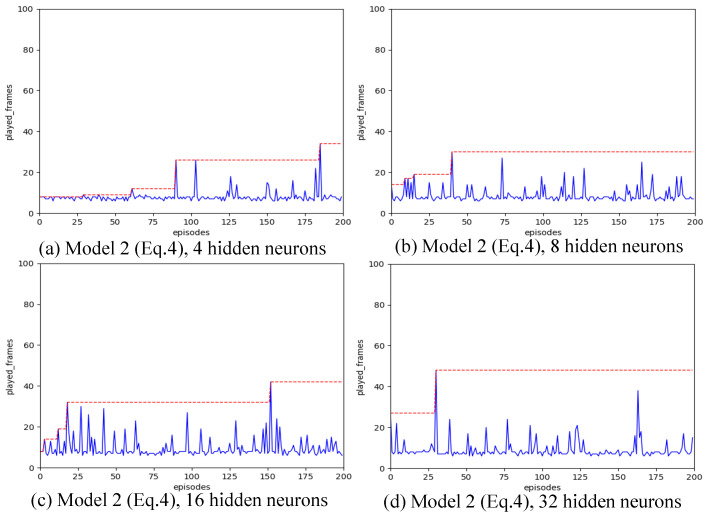
Result for Model 2 with different number of hidden neurons, after increasing the increasing the number of hidden layer neurons, the search power reinforcement learning algorithms increased slightly.

**Figure 6:**
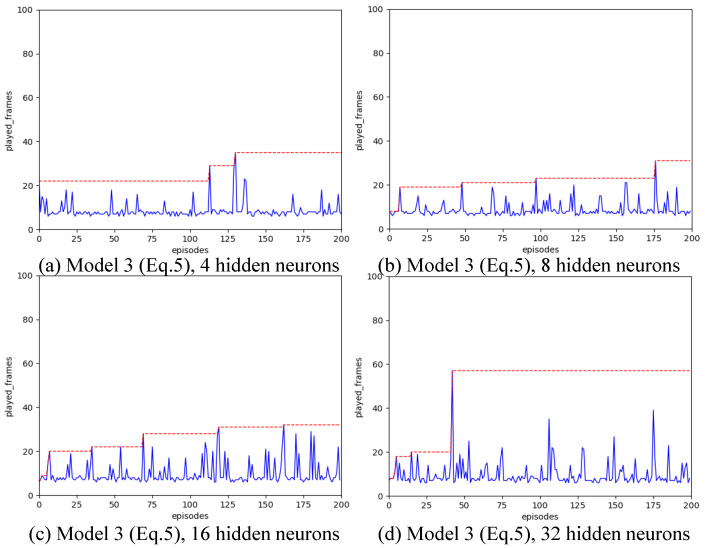
Result for Model 3 with different number of hidden neurons, as the number of hidden layer neurons increases, the search effect is significantly enhanced.

**Figure 7:**
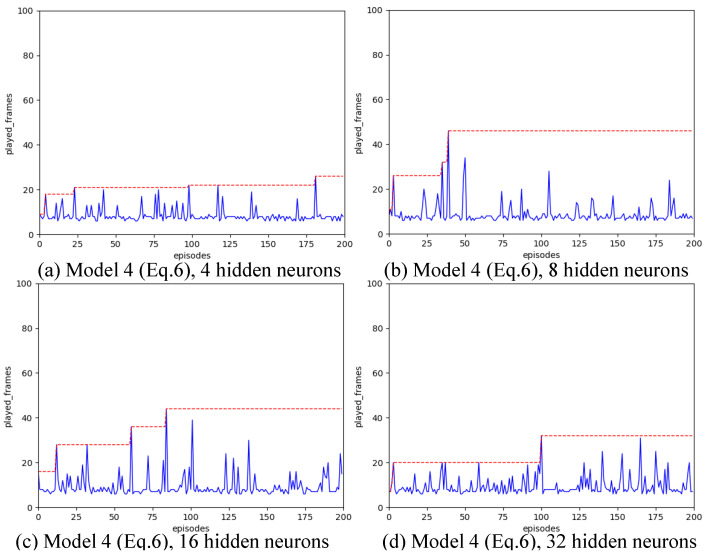
Result for Model 4 with different number of hidden neurons, after increasing the number of hidden layer neurons from 16 to 32, the search power of reinforcement learning algorithms has decreased.

**Figure 8:**
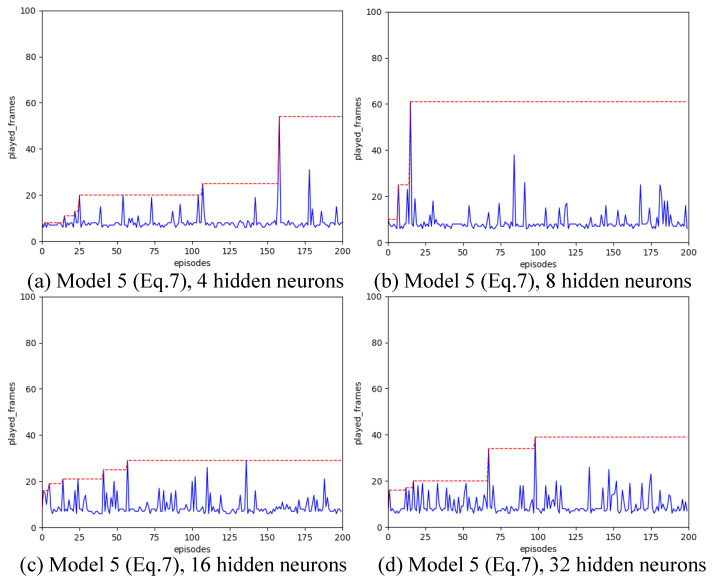
Result for Model 5 with different number of hidden neurons, achieving the most effective results when the number of hidden layer neurons is 8, and increasing the number of hidden layer neurons results in diminished effectiveness.

Strategy A regards each synapse as an agent. We have introduced various designs for state spaces. Our configuration includes a learning rate of α=0.01, a spiking simulation duration of T=16, and 200 iterations. We experimented with hidden layer neuron counts of 4, 8, 16, and 32 to assess the impact of different state space configurations. The outcomes are illustrated in Figure 4, Figure 5, Figure 6, Figure 7 and Figure 8, with the optimal result for each model (i.e., state space design) detailed in Table 2.

As shown in Figure 4, Figure 5, Figure 6, Figure 7 and Figure 8, the experimental results demonstrate that different state space designs significantly influence the performance of reinforcement learning algorithms.

When each model is examined individually, the following can be observed:

(1) Model 1 demonstrates relatively superior search performance when the hidden layer contains 8 neurons. The inverted pendulum achieves balance stability for approximately 60 frames;
(2) Model 2 determined the best strategy when the hidden layer was configured with 32 neurons. The inverted pendulum remained balanced for about 48 frames.
(3) Model 3 demonstrates optimal search performance when the hidden layer contains 32 neurons. The results show minimal differences when the hidden layer has 4, 8, or 16 neurons;
(4) Model 4 shows improved performance with a hidden layer consisting of either 8 or 16 neurons. Nevertheless, increasing the hidden layer’s neuron count to 32 results in a reduction in the model’s search performance.
(5) Model 5 shows enhanced performance when equipped with either 4 or 8 neurons in the hidden layer. Specifically, with 8 neurons, the inverted pendulum manages to maintain balance for about 62 frames. Yet, adding more neurons to the hidden layer reduces its effectiveness.

When the quantity of neurons in the hidden layer remains constant, yet the model differs, the following outcomes will be noted:

(1) With the number of neurons in the hidden layer set to 4, both Model 1 and Model 3 initially reached a peak before dropping to lower levels, suggesting a transition from a heuristic search to a more thorough search method. Conversely, Model 2 and Model 4 were ineffective in steering the weight updates within the spiking neural network. On the other hand, Model 5, being the most simplified by excluding the second-order Markov states, exhibited the best search performance. Remarkably, it showed a pronounced heuristic effect in subsequent searches after peaking.
(2) With the number of neurons in the hidden layer set to 8, the five models all show a certain degree of search effect;
(3) With the number of neurons in the hidden layer set to 16, none of the five models showed the optimal search effect;
(4) With the number of neurons in the hidden layer set to 32, Model 2 and Model 3 show the best results.

### 4.4. Result for Strategy B

In Strategy B, the SNN is regarded as an agent. The iteration period is established at 200. We assess the outcomes when the hidden layer’s neuron count is adjusted to 4, 8, 16, 32, and 64, respectively.

**Figure 9:**
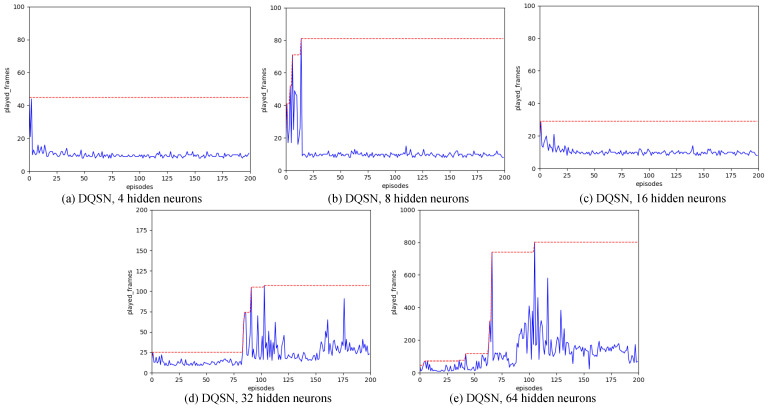
Result for strategy B with different number of hidden neurons, with the increasing of the number of hidden layer neurons, the search power of reinforcement learning algorithms increased obviously.

From the experimental results (shown in Figure 9), it is clear that the result of DQSN is not good when the number of hidden layer neurons is small. When the number of hidden layer neurons increases to 64, the strategy learning ability of DQSN is significantly improved.

### 4.5. Discussion

#### 4.5.1. For Strategy A

The experiments clearly show that variations in state space design significantly impact the effectiveness of the strategy A reinforcement learning training algorithm. Simply adding more neurons to the hidden layers does not necessarily enhance the SNN’s capability to learn the inverted pendulum balance strategy. Specifically, in models 1, 4, and 5, when the number of hidden layer neurons was increased from 8 to 32, the effectiveness of the reinforcement learning algorithms actually diminished.

According to our analysis, the reasons might be:

- The model employs a single-step update strategy, meaning that an excessive number of hidden layer neurons, like 16 or 32, can cause reinforcement learning to process a lot of irrelevant information during the early stages of training. This can disrupt the DQN’s ability to memorize and choose high-reward actions. The SNN functions as a cohesive unit. When using the ϵ-greedy strategy to navigate the state space, optimizing some parts of the network might simultaneously cause degradation in others. Consequently, increasing the hidden layer neurons might lower the chances of selecting actions that enhance the overall neural network, thereby decreasing the efficiency of the reinforcement learning training algorithm.
- Model 1’s state space design exclusively accounts for past Actions and their associated Rewards, without factoring in synaptic weights or spatial information. It depends entirely on second-order Markov equations for reinforcement learning, which might lead to substantial information loss in extensive networks. If this observation is valid, it implies that in large-scale networks where various segments might be tasked with different functions, differentiation is necessary. In such scenarios, topological information becomes an essential factor during the learning process.
- It is possible that the single-step update strategy is not efficient. For future work, we intend to employ the Monte Carlo update method rather than the single-step update method.

To sum up, Model 4, which integrates all synaptic weight updates into the state space, shows poor training performance when the hidden layer has too many neurons. This leads to inefficient training, high computational costs, and reduced model effectiveness. Potential reasons include: (1) The state space of Model 4 is too expansive, and the neural network’s architecture doesn’t have enough representational ability to accurately model each state’s values within this large space; (2) Not enough training iterations; (3) Inadequately calibrated ϵ values in the ϵ-greedy strategy; (4) An abundance of invalid data in the experience replay mechanism that overwrites valid information. Suggested improvements are: (1) consider adding more layers and hidden neurons to the neural network; (2) try increasing the number of training iterations; (3) experiment with various ϵ value configurations to test performance.

Conversely, Model 5, which features the most straightforward state space design (comprising solely the current synaptic weight and the synapse’s topological position information), retains excellent search abilities (achieving the most frames for Strategy A of the inverted pendulum) even with a minimal number of hidden layer neurons. Hence, it may not be necessary for the state space to be overly complex. We suggest this is because Model 5’s state space design exclusively includes synaptic weights and topology as represented by positional information. When synaptic weights are adjusted according to policy-directed actions, the system emphasizes the topological context of each agent (i.e., each synapse). This process is similar to how different brain regions carry out specialized functions, allowing agents in various locations to develop distinct capabilities and structures. As a result, the model attains superior learning outcomes.

Meanwhile, Model 1, which solely relies on second-order Markov equations, produces the longest-lasting synaptic update strategy during training.

Overall, the comparison among the five models reveals that both Model 5, which has 4 hidden neurons, and Model 1, with 8 hidden neurons, surpass the other models and offer significant room for optimization. These two models provide a foundation for additional research.

#### 4.5.2. For Strategy B

The performance of the DQSN algorithm is suboptimal when there are 4 or 16 neurons in the hidden layer. However, as the number of neurons in the hidden layer increases, the search capabilities of the DQSN-based reinforcement learning algorithm become more apparent. When the hidden layer contains 64 neurons, the DQSN is capable of effectively training reinforcement learning algorithms to sustain an inverted pendulum in balance for more than 100 frames, with the highest performance achieving nearly 800 frames of stable equilibrium.

It is clear that the BP-based DQSN (Strategy B) offers notable advantages in training efficiency and model performance when compared to the gradient-free reinforcement learning approach (Strategy A). However, Strategy A is more aligned with biological plausibility because it considers biological characteristics and factors when designing the state space. We propose that biological features such as the differentiation of biological structures and the small-world properties of topological connections could be valuable avenues for enhancing Strategy A. By crafting an appropriate state space, the potential of Strategy A can be greatly enhanced.

## 5. Conclusions

In this swork, we have investigated how to incorporate reinforcement learning strategies into the training process for SNN learning, considering neural network dynamics. We proposed and assessed two different strategies. Strategy A views a synapse as an agent. In designing the state space for Strategy A, we evaluated the effect of different combinations of the prior action, related reward, current synaptic weight, and synapse’s topological location. The results indicate that the state space design significantly impacts the reinforcement learning’s effectiveness. Strategy B regards the SNN as an agent. Our introduced DQSN system adopts a DQN-like architecture, substituting DNN with SNN, performing error backpropagation through gradient substitution, and utilizing methods such as the ϵ-greedy strategy, single-step updates, and experience replay.

Despite the fact that DQSN (Strategy B) is currently outperforming Strategy A, we are confident that Strategy A has more yet-to-be-realized potential. The reason is that Strategy A can more effectively utilize topological differences to enhance differential learning, and with ongoing state space optimization, it will reveal even more value. This is the future direction we intend to pursue.

Our research demonstrates that applying reinforcement learning methods to update parameters and weights in SNN subunits requires substantial computational time and resources. Therefore, when implementing reinforcement learning, it is crucial to design the state space, action space, and SNN architecture appropriately. It is essential to optimize model performance while minimizing training costs. The comparative experiments in this paper also show that the relatively simple state space design scheme and the SNN structure can achieve better results under the background of relatively limited computing power.

Another reason we prefer Strategy A is that its state space design incorporates neural dynamics, which aids in causal-based learning and enhances interpretability. The causal learning mechanism is fundamental to how the human brain learns, rooted in its dynamics. In our research, by integrating neural network dynamics into the reinforcement learning framework, we can infuse a causal learning mechanism into the SNN learning process. We hold the view that causal learning is crucial for developing interpretable AI, and consequently, it is also vital for creating trustworthy AI.

In upcoming research, we plan to integrate the adaptive growth of the SNN architecture and align it with neuronal dynamics to create a more robust reinforcement learning-based approach for SNN training. Our goal is to investigate and uncover the mechanisms connecting neurons in order to enhance our understanding and interpretation of the SNN learning process and mechanisms. Overall, the integration of reinforcement learning with SNN holds significant potential value for advancing trustworthy AI research.

## Figures and Tables

**Table 1:** Parameters of LIF model.

Parameters	Description	Unit	Set to
τ	Membrane time constant	ms	2.0
$V_{th}$	Spike threshold	mV	1.0
$V_{reset}$	Reset potential of the membrane	mV	0.0

**Table 2:** Best results in played frames of different state space designs for Strategy A.

No. of Hidden Layer Neurons	Model 1	Model 2	Model 3	Model 4	Model 5
4	22	34	35	26	57
8	60	30	30	44	62
16	32	42	32	42	32
32	38	48	59	32	41

## Data Availability

The data presented in this study are available on request from the corresponding author.
